# Effect of Denosumab Compared With Risedronate on Bone Strength in Patients Initiating or Continuing Glucocorticoid Treatment

**DOI:** 10.1002/jbmr.4551

**Published:** 2022-04-19

**Authors:** Piet Geusens, Melissa SAM Bevers, Bert van Rietbergen, Osvaldo D Messina, Eric Lespessailles, Beatriz Oliveri, Roland Chapurlat, Klaus Engelke, Arkadi Chines, Shuang Huang, Kenneth G Saag, Joop P van den Bergh

**Affiliations:** ^1^ Department of Internal Medicine Subdivision of Rheumatology, Maastricht University Medical Center Maastricht The Netherlands; ^2^ Department of Medicine and Life Sciences Hasselt University Hasselt Belgium; ^3^ Department of Internal Medicine VieCuri Medical Center Venlo The Netherlands; ^4^ NUTRIM School for Nutrition and Translational Research in Metabolism Maastricht University Medical Center Maastricht The Netherlands; ^5^ Department of Biomedical Engineering Eindhoven University of Technology Eindhoven The Netherlands; ^6^ Department of Orthopedic Surgery Maastricht University Medical Center Maastricht The Netherlands; ^7^ IRO Medical Center Investigaciones Reumatologicas y Osteologicas SRL Buenos Aires Argentina; ^8^ Regional Hospital of Orleans Translational Medicine Research Platform, PRIMMO Orleans France; ^9^ INIGEM, Hospital de Clínicas José de San Martin Buenos Aires Argentina; ^10^ INSERM UMR 1033 Université de Lyon, Hospital Edouard Herriot Lyon France; ^11^ Bioclinica Inc Hamburg Germany; ^12^ Department of Medicine 3 FAU University Erlangen‐Nürnberg and Universitätsklinikum Erlangen Erlangen Germany; ^13^ Amgen Inc Thousand Oaks CA USA; ^14^ University of Alabama at Birmingham Birmingham AL USA

**Keywords:** GLUCOCORTICOID‐INDUCED OSTEOPOROSIS, DENOSUMAB, RISEDRONATE, BONE STRENGTH, HIGH‐RESOLUTION PERIPHERAL QUANTITATIVE COMPUTED TOMOGRAPHY (HR‐pQCT)

## Abstract

In a randomized clinical trial in patients initiating glucocorticoid therapy (GC‐I) or on long‐term therapy (GC‐C), denosumab every 6 months increased spine and hip bone mineral density at 12 and 24 months significantly more than daily risedronate. The aim of this study was to evaluate the effects of denosumab compared with risedronate on bone strength and microarchitecture measured by high‐resolution peripheral quantitative computed tomography (HR‐pQCT) in GC‐I and GC‐C. A subset of 110 patients had high‐resolution peripheral quantitative computed tomography (HR‐pQCT) scans of the distal radius and tibia at baseline and at 12 and 24 months. Cortical and trabecular microarchitecture were assessed with standard analyses and failure load (FL) with micro‐finite element analysis. At the radius at 24 months, FL remained unchanged with denosumab and significantly decreased with risedronate in GC‐I (−4.1%, 95% confidence interval [CI] −6.4, −1.8) and, in GC‐C, it significantly increased with denosumab (4.3%, 95% CI 2.1, 6.4) and remained unchanged with risedronate. Consequently, FL was significantly higher with denosumab than with risedronate in GC‐I (5.6%, 95% CI 2.4, 8.7, *p* < 0.001) and in GC‐C (4.1%, 95% CI 1.1, 7.2, *p* = 0.011). We also found significant differences between denosumab and risedronate in percentage changes in cortical and trabecular microarchitectural parameters in GC‐I and GC‐C. Similar results were found at the tibia. To conclude, this HR‐pQCT study shows that denosumab is superior to risedronate in terms of preventing FL loss at the distal radius and tibia in GC‐I and in increasing FL at the radius in GC‐C, based on significant differences in changes in the cortical and trabecular bone compartments between treatment groups in GC‐I and GC‐C. These results suggest that denosumab could be a useful therapeutic option in patients initiating GC therapy or on long‐term GC therapy and may contribute to treatment decisions in this patient population. © 2022 The Authors. *Journal of Bone and Mineral Research* published by Wiley Periodicals LLC on behalf of American Society for Bone and Mineral Research (ASBMR).

## Introduction

Patients who use glucocorticoids (GC) are at increased risk of vertebral and non‐vertebral fractures, immediately when they initiate (GC‐I) or continue treatment (GC‐C).^(^ ^1^, ^2^, ^3^ ^)^ In a randomized phase 3 clinical trial, gains in areal bone mineral density (aBMD) by dual‐energy X‐ray absorptiometry (DXA) were greater with denosumab than with risedronate at the lumbar spine, femoral neck, total hip, and radius at 12 and 24 months of treatment in both GC‐I and GC‐C,^(^ ^4^, ^5^ ^)^ but that study did not provide insights into changes in cortical and trabecular microarchitecture or bone strength.

High‐resolution peripheral quantitative computed tomography (HR‐pQCT) allows assessment of bone microarchitecture and strength to evaluate disease and treatment effects.^(^ ^6^ ^)^ Earlier cross‐sectional case–control studies using HR‐pQCT reported disturbed cortical and trabecular microarchitecture and decreased bone stiffness at the distal radius and tibia in patients on long‐term oral or inhaled GC,^(^ ^7^, ^8^ ^)^ but data on the effects of treatment on bone microarchitecture and strength in GC‐induced osteoporosis (GIOP) are lacking. In postmenopausal women, positive effects have been described with denosumab and risedronate on bone microarchitecture using HR‐pQCT^(^ ^9^, ^10^, ^11^ ^)^ and with denosumab on bone strength using QCT.^(^ ^9^, ^12^, ^13^ ^)^ However, the pathophysiology of bone loss in GIOP is different from postmenopausal osteoporosis (PMOP),^(^ ^14^, ^15^, ^16^, ^17^, ^18^, ^19^ ^)^ and thus the treatment effects of denosumab and risedronate on bone microarchitecture and strength in PMOP cannot be extrapolated to GIOP. Therefore, and in view of the differences in the effects on aBMD between denosumab and risedronate in GC‐I and GC‐C,^(^ ^4^, ^5^ ^)^ the aim of this study was to evaluate changes in cortical and trabecular microarchitecture and estimated bone strength assessed with HR‐pQCT during 24 months with denosumab compared with risedronate in GC‐I and GC‐C.

## Subjects and Methods

### Study population and design

The study population of this HR‐pQCT study included a subgroup of GC‐treated patients enrolled in a phase 3, double‐blind, double‐dummy, and active‐controlled clinical trial on the effects of denosumab compared with risedronate on aBMD (NCT01575873). Study population and design of this multicenter study have previously been described in detail.^(^ ^4^, ^5^ ^)^ The study was conducted in accordance with the Declaration of Helsinki and followed the International Conference for Harmonization Guidelines for Good Clinical Practice. An independent review board approved the study design for each center. Written informed consent was obtained from each patient before study participation.

### HR‐pQCT imaging

HR‐pQCT scans were taken from patients who consented to participate in this substudy and who were treated in centers with access to an HR‐pQCT scanner. This included a total of 110 (56 denosumab, 54 risedronate) of the 590 GC‐treated patients participating in the main study. The HR‐pQCT scans in all participating centers were acquired using the first‐generation XtremeCT scanner (XtremeCT, Scanco Medical AG, Bruttisellen, Switzerland), and none of the centers switched to the second‐generation scanner during study duration. All centers applied the same scan protocol using the default clinical settings defined by the manufacturer of the scanner to scan a standardized region of the distal radius and tibia.^(^ ^20^ ^)^ Additionally, cross‐validation was performed using a dedicated phantom (QRM Forearm QC phantom, QRM GmbH, Mohrendorf, Germany) that was scanned at all study sites. This phantom consisted of a European forearm phantom coupled to a QRM calibration phantom, which allowed for correction of potential X‐ray field inhomogeneities.

Scan‐quality assessment and analyses were performed centrally. Baseline and follow‐up scans were registered using standard automatic two‐dimensional slice‐matching, followed by analysis of the common bone volume of interest according to the standard evaluation protocol.^(^ ^20^, ^21^ ^)^ Additionally, bone strength was estimated in terms of failure load (FL) and bone stiffness using standard linear‐elastic micro‐finite element (μFE) analysis.^(^ ^20^, ^22^ ^)^ If the common volume (ie, number of slices overlapping at all three visits) was <60, the radius or tibia scans of all visits of the corresponding patient were excluded from analysis. Cortical assessments were not performed for movement artifacts of grade 2 or higher on a scale of 1 (no movement) to 4 (severe movement).^(^ ^23^ ^)^

### Statistical analysis

Repeated‐measures mixed‐effects models were used to estimate the percentage changes from baseline in each treatment group. Consistent with the main study,^(^ ^4^, ^5^ ^)^ the models were adjusted for treatment (main effect) and baseline value (covariate). Duration of prior GC‐use (<12 months versus ≥12 months) was an additional covariate in the models for GC‐C. Percentage changes were expressed as least‐square means with 95% confidence intervals (95% CI). Additionally, the difference between the least‐square means of denosumab and risedronate was estimated and expressed as least‐square means with 95% CI and *p* value (significance level α = 0.05; not adjusted for multiple comparisons).

## Results

Baseline characteristics were balanced between the two treatment groups within the GC‐I and GC‐C subpopulations, except for lumbar spine *T*‐score in GC‐C (Tables 1 and 2). Because of insufficient slice overlap among visits (<60 slices), radius scans from three patients and tibia scans from one patient were excluded from analysis. Common number of slices was >80 for all patients except for five patients at the radius and two patients at the tibia (60 to 80 slices). Additionally, the radius scans of one patient with an old radius fracture and one patient with inconsistent laterality of follow‐up compared with baseline were excluded from analysis. The data of another patient were excluded for being an outlier due to the near absence of trabecular bone. For five visits, cortical parameters were not assessed in the radius because of movement artifacts.

**Table 1:** Baseline Characteristics

	GC‐initiating		GC‐continuing	
	Risedronate (*N* = 24)	Denosumab (*N* = 32)	Risedronate (*N* = 30)	Denosumab (*N* = 24)
Female sex	15 (62.5)	19 (59.4)	28 (93.3)	19 (79.2)
Premenopause	0 (0.0)	1 (5.3)	0 (0.0)	1 (5.3)
Postmenopause	15 (100.0)	18 (94.7)	28 (100.0)	18 (94.7)
Age (years)	66.0 ± 13.1	68.5 ± 9.8	64.2 ± 8.8	63.6 ± 9.8
Race				
White	24 (100.0)	32 (100.0)	30 (100.0)	23 (95.8)
Asian	0 (0.0)	0 (0.0)	0 (0.0)	0 (0.0)
Black or African American	0 (0.0)	0 (0.0)	0 (0.0)	1 (4.2)
Other	0 (0.0)	0 (0.0)	0 (0.0)	0 (0.0)
Baseline daily GC dose (mg)^a^	18.91 ± 9.91	21.41 ± 15.68	10.32 ± 6.52	11.33 ± 4.95
Duration of prior GC use				
<12 months	24 (100.0)	32 (100.0)	4 (13.3)	4 (16.7)
≥12 months	0 (0.0)	0 (0.0)	26 (86.7)	20 (83.3)
BMD *T*‐score (DXA)				
Lumbar spine	−0.77 ± 1.83^b^	−0.98 ± 1.95	−2.60 ± 1.08	−1.64 ± 1.80
Total hip	−0.77 ± 0.79	−1.15 ± 0.97^b^	−1.73 ± 0.86	−1.62 ± 0.85

GC = glucocorticoid; BMD = bone mineral density; DXA = dual‐energy X‐ray absorptiometry.

Data are reported as *n* (%) or mean ± standard deviation.

^a^ Dose in prednisone equivalents.

^b^ Assessed in N‐1 individuals.

**Table 2:** Baseline Characteristics of HR‐pQCT Measurements in Patients Initiating Glucocorticoid Therapy (GC‐I) or on Long‐Term Therapy (GC‐C) at the Distal Radius and Tibia

	GC‐initiating		GC‐continuing	
	Risedronate (*N* = 24)	Denosumab (*N* = 32)	Risedronate (*N* = 30)	Denosumab (*N* = 24)
Distal radius	*n* = 24	*n* = 29	*n* = 21	*n* = 20
μFE				
Stiffness (kN/mm)	77.8 ± 28.6^a^	75.3 ± 33.4^b^	49.7 ± 9.81^e^	59.6 ± 23.1^f^
FL (kN)	3.94 ± 1.40^a^	3.78 ± 1.64^b^	2.51 ± 0.50^e^	2.98 ± 1.17^f^
Total bone				
Total volume (mm^3^)	2842.5 ± 639.1^c^	3060.5 ± 822.7	2538.6 ± 447.2^g^	2603.6 ± 658.4
Tt.BMD (mg HA/cm^3^)	279.2 ± 75.8	267.5 ± 69.5	213.5 ± 54.3	236.0 ± 70.0
Cortical bone				
Cortical volume (mm^3^)	522.5 ± 148.9^c^	535.8 ± 162.7	416.3 ± 71.4^g^	458.5 ± 98.3
Ct.BMD (mg HA/cm^3^)	792.1 ± 69.4	790.7 ± 77.3	787.4 ± 48.4	777.5 ± 104.8
Ct.Th (mm)	0.68 ± 0.23	0.67 ± 0.24	0.53 ± 0.13	0.58 ± 0.23
Ct.Po (%)	2.55 ± 1.12^c^	2.75 ± 1.24	2.67 ± 1.10^g^	2.83 ± 1.34^h^
Trabecular bone				
Tb.BMD (mg HA/cm^3^)	149.1 ± 49.7	140.0 ± 46.8	95.2 ± 42.9	106.6 ± 50.3
Tb.BV/TV (−)	0.12 ± 0.04	0.12 ± 0.04	0.08 ± 0.04	0.09 ± 0.04
Tb.N (mm^−1^)	1.78 ± 0.37	1.71 ± 0.39	1.36 ± 0.44	1.44 ± 0.47
Tb.Th (mm)	0.07 ± 0.01	0.07 ± 0.01	0.06 ± 0.01	0.06 ± 0.01
Tb.Sp (mm)	0.52 ± 0.15	0.56 ± 0.21	0.79 ± 0.40	0.72 ± 0.30

Distal tibia	n = 24	n = 29	n = 24	n = 21
μFE				
Stiffness (kN/mm)	197.8 ± 56.3^d^	194.4 ± 65.5^b^	134.4 ± 27.6^h^	155.9 ± 37.8^e^
FL (kN)	9.99 ± 2.76^d^	9.85 ± 3.20^b^	6.90 ± 1.44^h^	7.95 ± 1.83^e^
Total bone				
Total volume (mm^3^)	6866.2 ± 1096.0^a^	6963.0 ± 1315.9	6462.9 ± 1013.9	6345.7 ± 911.2
Tt.BMD (mg HA/cm^3^)	259.5 ± 69.7	261.7 ± 55.3	**192.8 ± 41.0**	**232.2 ± 53.4**
Cortical bone				
Cortical volume (mm^3^)	1048.5 ± 319.8^a^	1085.5 ± 347.2	**803.1 ± 140.5**	**925.5 ± 140.4**
Ct.BMD (mg HA/cm^3^)	805.3 ± 61.6	795.1 ± 60.9	793.5 ± 61.2	800.2 ± 88.2
Ct.Th (mm)	1.03 ± 0.37	1.04 ± 0.35	**0.73 ± 0.14**	**0.89 ± 0.27**
Ct.Po (%)	7.16 ± 1.75^a^	7.43 ± 2.12	7.40 ± 3.18	7.60 ± 2.88
Trabecular bone				
Tb.BMD (mg HA/cm^3^)	151.3 ± 42.2	155.1 ± 32.3	106.3 ± 41.4	127.6 ± 45.8
Tb.BV/TV (−)	0.13 ± 0.04	0.13 ± 0.03	0.09 ± 0.03	0.11 ± 0.04
Tb.N (mm^−1^)	1.72 ± 0.38	1.81 ± 0.31	1.44 ± 0.40	1.63 ± 0.46
Tb.Th (mm)	0.07 ± 0.01	0.07 ± 0.01	0.06 ± 0.01	0.07 ± 0.01
Tb.Sp (mm)	0.53 ± 0.13	0.50 ± 0.10	0.68 ± 0.19	0.62 ± 0.32

μFE = micro‐finite element; FL = failure load; Tt.BMD = total bone mineral density; Ct.BMD = cortical bone mineral density; Ct.Th = cortical thickness; Ct. Po = cortical porosity; Tb.BMD = trabecular bone mineral density; Tb.BV/TV = trabecular bone volume fraction; Tb.N = trabecular number; Tb.Th = trabecular thickness; Tb.Sp = trabecular separation.

Data are reported as mean ± standard deviation.

*N* = number of randomized patients enrolled in the HR‐pQCT substudy; *n* = number of patients with observed data.

^a^ *n* = 22.

^b^ *n* = 25.

^c^ *n* = 23.

^d^ *n* = 21.

^e^ *n* = 15.

^f^ *n* = 14.

^g^ *n* = 20.

^h^ *n* = 19.

Significant differences between risedronate and denosumab are reported in bold (two‐sample independent *t* test; *p* < 0.05).

### Evaluation of estimated bone strength

**Fig. 1:**
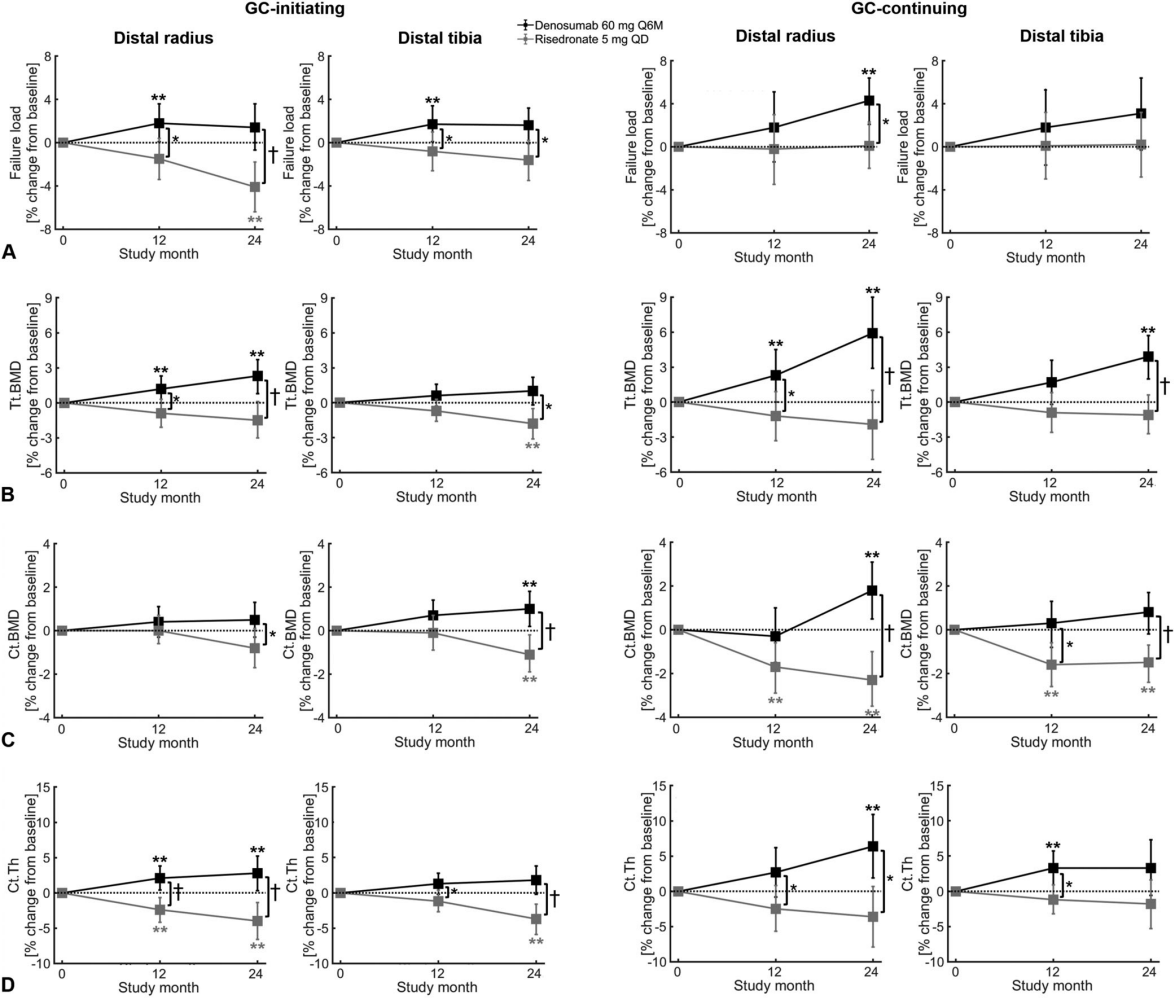
Percentage changes from baseline in bone failure load (*A*), total bone mineral density (Tt.BMD) (*B*), cortical BMD (Ct.BMD) (*C*), and cortical thickness (Ct.Th) (*D*) at the distal radius and tibia with 60 mg of subcutaneous denosumab every 6 months or 5 mg of oral risedronate daily in patients initiating (<3 months) or continuing glucocorticoid treatment (≥3 months). Significant changes within the treatment groups compared with baseline are denoted with ** (*p* < 0.05); significant changes between the treatment groups are denoted with * (*p* < 0.05) and † (*p* ≤ 0.001).

In GC‐I, FL significantly increased compared with baseline at 12 months with denosumab (radius: 1.8%, 95% CI 0.1, 3.6; tibia: 1.7%, 95% CI 0.1, 3.4) and did not change with risedronate (Table 3 and Fig. 1). This led to significant differences in percentage change between the two drugs (radius: 3.3%, *p* = 0.013; tibia: 2.5%, *p* = 0.043). At 24 months, FL remained unchanged with denosumab, while it significantly decreased compared with baseline with risedronate at the radius (−4.1%, 95% CI −6.4, −1.8). This resulted in a significant between‐treatment difference at the radius (5.6%, *p* < 0.001) and also at the tibia (3.2%, *p* = 0.013), although the changes at the tibia at 24 months did not reach significance. Similar results were found in stiffness.

**Table 3:** Percentage Change From Baseline in μFE‐Based Parameters Describing Bone Strength at the Distal Radius and Tibia in Patients Initiating Glucocorticoid Therapy (GC‐I) or on Long‐Term Therapy (GC‐C)

		Distal radius		Distal tibia	
		Month 12	Month 24	Month 12	Month 24
GC‐initiating		*n*_D_ = 25, *n*_R_ = 22	*n*_D_ = 23, *n*_R_ = 20	*n*_D_ = 25, *n*_R_ = 21	*n*_D_ = 23, *n*_R_ = 18
FL	DMAb (*N* = 32)	**1.8 (0.1, 3.6)**	1.4 (−0.7, 3.6)	**1.7 (0.1, 3.4)**	1.6 (−0.1, 3.2)
	RIS (*N* = 24)	−1.5 (−3.4, 0.4)	**−4.1 (−6.4, −1.8)**	−0.8 (−2.6, 1.0)	−1.6 (−3.5, 0.2)
	DMAb‐RIS	3.3 (0.7, 5.9)*	**5.6 (2.4, 8.7)†**	2.5 (0.1, 4.9)*	3.2 (0.7, 5.7)*
Stiffness	DMAb	1.5 (−0.3, 3.4)	0.9 (−1.3, 3.1)	**2.1 (0.1, 4.1)**	1.8 (−0.2, 3.7)
	RIS	−1.9 (−3.9, 0.1)	**−4.4 (−6.8, −2.0)**	−0.8 (−3.0, 1.5)	−1.6 (−3.8, 0.6)
	DMAb‐RIS	3.5 (0.8, 6.1)*	5.3 (2.0, 8.5)*	2.9 (−0.1, 5.9)	3.3 (0.4, 6.3)*

GC‐continuing		*n*_D_ = 14, *n*_R_ = 14	*n*_D_ = 12, *n*_R_ = 12	*n*_D_ = 14, *n*_R_ = 18	*n*_D_ = 13, *n*_R_ = 16
FL	DMAb (*N* = 24)	1.8 (−1.4, 5.1)	**4.3 (2.1, 6.4)**	1.8 (−1.7, 5.3)	3.1 (−0.3, 6.4)
	RIS (*N* = 30)	−0.2 (−3.5, 3.0)	0.1 (−2.0, 2.3)	0.1 (−3.0, 3.2)	0.2 (−2.8, 3.2)
	DMAb‐RIS	2.0 (−2.6, 6.7)	4.1 (1.1, 7.2)*	1.7 (−3.1, 6.5)	2.9 (−1.8, 7.5)
Stiffness	DMAb	1.2 (−2.2, 4.6)	**3.6 (1.6, 5.5)**	2.3 (−1.9, 6.4)	3.7 (−0.3, 7.6)
	RIS	−0.4 (−3.9, 3.0)	−0.1 (−2.0, 1.9)	0.2 (−3.4, 3.9)	0.1 (−3.4, 3.6)
	DMAb‐RIS	1.6 (−3.3, 6.6)	3.6 (0.9, 6.4)*	2.0 (−3.6, 7.7)	3.5 (−1.9, 8.9)

FL = failure load; DMAb = denosumab; RIS = risedronate.

Data are reported as least‐square means (95% confidence interval).

*N* = number of randomized patients enrolled in the HR‐pQCT substudy;

*n*_D_ = number of patients with observed data receiving denosumab; *n*_R_ = number of patients with observed data receiving risedronate.

Percentage changes with and differences between the treatment groups are based on repeated‐measures mixed‐effects models adjusted for treatment and baseline value (GC‐initiating) or treatment, baseline value, and duration of prior GC use (<12 months versus ≥12 months) (GC‐continuing). Significant changes within and differences between the treatment groups are reported in bold (*p* < 0.05). *p < 0.05; †*p* ≤ 0.001 for differences between the treatment groups.

In GC‐C, FL remained unchanged with denosumab and risedronate at 12 months and significantly increased with denosumab (radius: 4.3%, 95% CI 2.1, 6.4) and remained unchanged with risedronate at 24 months (Table 3 and Fig. 1). Corresponding difference between denosumab and risedronate at 24 months was significant (radius: 4.1%, *p* = 0.011). Changes and differences in stiffness were similar.

### Evaluation of bone volume

In GC‐I and GC‐C, significant changes with and differences between denosumab and risedronate were not found in total volume, but they were found in cortical volume (Tables 4 and 5). In GC‐I at 12 months, cortical volume significantly increased with denosumab (radius: 1.8%, 95% CI 0.3, 3.3; tibia: 1.0%, 95% CI 0.0, 2.0) and remained unchanged with risedronate. Consequently, the differences between denosumab and risedronate were significant (radius: 3.0%, *p* = 0.008; tibia: 1.8%, *p* = 0.021). At 24 months, it significantly changed at the radius with denosumab (2.9%, 95% CI 1.4, 4.5) and risedronate (−2.1%, 95% CI −3.7, −0.4) with corresponding significant between‐treatment difference (5.0%, *p* < 0.001). Although the change at the tibia at 24 months did not reach significance, corresponding between‐treatment difference did (2.5%, *p* = 0.028). In GC‐C, cortical volume remained unchanged with denosumab and risedronate at 12 months. At 24 months, it significantly increased with denosumab (radius: 3.1%, 95% CI 0.5, 5.6; tibia: 5.0%, 95% CI 1.8, 8.1) and decreased with risedronate (tibia: −2.9%, 95% CI −5.7, −0.1), resulting in significant between‐treatment differences (radius: 4.8%, *p* = 0.013; tibia: 7.9%, *p* = 0.001).

**Table 4:** Percentage Change From Baseline in Parameters Describing Bone Volume, Density, and Microarchitecture of the Distal Radius and Tibia in Patients Initiating Glucocorticoid Therapy (GC‐I)

		Distal radius		Distal tibia	
		Month 12 (*n*_D_ = 24, *n*_R_ = 23)	Month 24 (*n*_D_ = 24, *n*_R_ = 21)	Month 12 (*n*_D_ = 25, *n*_R_ = 23)	Month 24 (*n*_D_ = 24, *n*_R_ = 20)
Total volume	DMAb (*N* = 32)	−1.3 (−2.9, 0.2)	−0.9 (−2.4, 0.6)^b^	−0.2 (−0.8, 0.3)	−0.1 (−0.6, 0.5)^d^
	RIS (*N* = 24)	−0.4 (−2.0, 1.2)^a^	0.6 (−1.1, 2.2)^b^	−0.2 (−0.8, 0.3)^c^	−0.5 (−1.1, 0.2)^d^
	DMAb‐RIS	−1.0 (−3.2, 1.2)	−1.5 (−3.7, 0.7)	−0.0 (−0.8, 0.7)	0.4 (−0.4, 1.2)
Tt.BMD	DMAb	**1.2 (0.0, 2.3)**	**2.3 (0.8, 3.7)**	0.6 (−0.3, 1.6)	1.0 (−0.2, 2.2)
	RIS	−0.9 (−2.1, 0.3)	−1.5 (−3.0, 0.1)	−0.7 (−1.6, 0.3)	**−1.8 (−3.1, −0.5)**
	DMAb‐RIS	2.1 (0.4, 3.8)*	**3.7 (1.6, 5.8)†**	1.3 (−0.1, 2.6)	2.9 (1.1, 4.6)*
Cortical volume	DMAb	**1.8 (0.3, 3.3)**	**2.9 (1.4, 4.5)** ^b^	**1.0 (0.0, 2.0)**	1.0 (−0.5, 2.5)^d^
	RIS	−1.2 (−2.8, 0.4)^a^	**−2.1 (−3.7, −0.4)** ^b^	−0.8 (−1.9, 0.3)^c^	−1.5 (−3.2, 0.1)^d^
	DMAb‐RIS	3.0 (0.8, 5.2)*	**5.0 (2.7, 7.3)†**	1.8 (0.3, 3.3)*	2.5 (0.3, 4.8)*
Ct.BMD	DMAb	0.4 (−0.3, 1.1)	0.5 (−0.3, 1.3)	0.7 (−0.1, 1.4)	**1.0 (0.2, 1.8)**
	RIS	0.0 (−0.6, 0.7)	−0.8 (−1.7, 0.1)	−0.1 (−0.9, 0.7)	**−1.1 (−1.9, −0.2)**
	DMAb‐RIS	0.4 (−0.6, 1.3)	1.4 (0.2, 2.5)*	0.7 (−0.4, 1.8)	**2.1 (0.9, 3.2)†**
Ct.Th	DMAb	**2.1 (0.4, 3.8)**	**2.8 (0.3, 5.2)**	1.3 (−0.2, 2.8)	1.8 (−0.2, 3.8)
	RIS	**−2.4 (−4.2, −0.7)**	**−4.0 (−6.6, −1.4)**	−1.2 (−2.7, 0.4)	**−3.7 (−5.9, −1.6)**
	DMAb‐RIS	**4.6 (2.1, 7.0)†**	**6.7 (3.2, 10.3)†**	2.5 (0.3, 4.7)*	**5.5 (2.6, 8.5)†**
Ct.Po	DMAb	1.9 (−4.6, 8.3)	**12.5 (4.1, 21.0)** ^b^	2.7 (−2.5, 7.9)	1.4 (−2.6, 5.5)^d^
	RIS	3.4 (−3.4, 10.2)^a^	8.1 (−1.0, 17.1)^b^	1.6 (−4.0, 7.3)^c^	**6.2 (1.6, 10.8)** ^d^
	DMAb‐RIS	−1.5 (−10.9, 7.9)	4.5 (−7.9, 16.9)	1.1 (−6.7, 8.8)	−4.7 (−10.9, 1.5)
Tb.BMD	DMAb	1.3 (−0.2, 2.8)	**3.4 (1.5, 5.3)**	0.5 (−0.3, 1.3)	0.8 (−0.5, 2.0)
	RIS	−0.0 (−1.6, 1.5)	0.1 (−1.9, 2.1)	0.1 (−0.7, 1.0)	−0.5 (−1.8, 0.9)
	DMAb‐RIS	1.3 (−0.8, 3.5)	3.3 (0.5, 6.0)*	0.4 (−0.8, 1.5)	1.2 (−0.6, 3.1)
Tb.BV/TV	DMAb	1.5 (−0.1, 3.1)	**3.8 (1.8, 5.7)**	0.8 (−0.1, 1.7)	**1.3 (0.0, 2.7)**
	RIS	0.4 (−1.3, 2.0)	1.0 (−1.1, 3.1)	0.7 (−0.3, 1.6)	0.4 (−1.0, 1.9)
	DMAb‐RIS	1.1 (−1.1, 3.4)	2.8 (−0.1, 5.7)	0.1 (−1.2, 1.4)	0.9 (−1.1, 2.9)
Tb.N	DMAb	2.7 (−0.7, 6.1)	**6.3 (2.3, 10.2)**	−0.8 (−4.5, 3.0)	2.2 (−1.8, 6.3)
	RIS	3.2 (−0.3, 6.6)	3.8 (−0.4, 8.0)	0.1 (−3.8, 4.0)	0.5 (−3.9, 4.9)
	DMAb‐RIS	−0.5 (−5.3, 4.4)	2.4 (−3.3, 8.2)	−0.8 (−6.3, 4.6)	1.7 (−4.3, 7.7)
Tb.Th	DMAb	−1.0 (−4.0, 2.0)	−1.5 (−4.3, 1.3)	2.8 (−1.0, 6.6)	0.2 (−3.4, 3.7)
	RIS	−1.8 (−4.8, 1.3)	−2.5 (−5.5, 0.5)	0.9 (−3.0, 4.8)	0.1 (−3.8, 4.0)
	DMAb‐RIS	0.8 (−3.5, 5.1)	1.0 (−3.1, 5.1)	1.9 (−3.6, 7.4)	0.0 (−5.2, 5.3)
Tb.Sp	DMAb	−2.4 (−5.9, 1.0)	**−5.8 (−9.7, −2.0)**	1.6 (−2.1, 5.4)	−1.3 (−5.1, 2.5)
	RIS	−2.4 (−5.9, 1.1)	−2.7 (−6.9, 1.4)	0.5 (−3.4, 4.4)	−0.2 (−4.4, 3.9)
	DMAb‐RIS	−0.1 (−5.0, 4.9)	−3.1 (−8.7, 2.6)	1.1 (−4.3, 6.6)	−1.1 (−6.7, 4.6)

DMAb = denosumab; RIS = risedronate; Tt.BMD = total bone mineral density; Ct.BMD = cortical bone mineral density; Ct.Th = cortical thickness; Ct. Po = cortical porosity; Tb.BMD = trabecular bone mineral density; Tb.BV/TV = trabecular bone volume fraction; Tb.N = trabecular number; Tb.Th = trabecular thickness; Tb.Sp = trabecular separation.

Data are reported as least‐square means (95% confidence interval).

*N* = number of randomized patients enrolled in the HR‐pQCT substudy; *n*_D_ = number of patients with observed data receiving denosumab; *n*_R_ = number of patients with observed data receiving risedronate.

^a^ *n*_R_ = 22.

^b^ *n*_R_ = 20 and *n*_D_ = 23.

^c^ *n*_R_ = 21.

^d^ *n*_R_ = 18 and *n*_D_ = 23.

Percentage changes with and differences between the treatment groups are based on repeated‐measures mixed‐effects models adjusted for treatment and baseline value. Significant changes within and differences between the treatment groups are reported in bold (*p* < 0.05). *p < 0.05 and †*p* ≤ 0.001 for differences between the treatment groups.

**Table 5:** Percentage Change From Baseline in Parameters Describing Bone Volume, Density, and Microarchitecture of the Distal Radius and Tibia in Patients on Long‐Term Glucocorticoid Therapy (GC‐C)

		Distal radius		Distal tibia	
		Month 12 (*n*_D_ = 14, *n*_R_ = 16)	Month 24 (*n*_D_ = 12, *n*_R_ = 13)	Month 12 (*n*_D_ = 15, *n*_R_ = 19)	Month 24 (*n*_D_ = 13, *n*_R_ = 16)
Total volume	DMAb (*N* = 24)	0.9 (−1.3, 3.1)	−0.2 (−2.0, 1.6)	−0.0 (−0.8, 0.8)^a^	−0.3 (−1.4, 0.7)
	RIS (*N* = 30)	0.4 (−1.8, 2.6)^a^	0.1 (−1.7, 1.9)^b^	0.5 (−0.3, 1.2)^e^	0.3 (−0.7, 1.2)
	DMAb‐RIS	0.5 (−2.7, 3.6)	−0.3 (−2.9, 2.2)	−0.5 (−1.6, 0.6)	−0.6 (−2.0, 0.8)
Tt.BMD	DMAb	**2.3 (0.0, 4.5)**	**5.9 (2.9, 9.0)**	1.7 (−0.2, 3.6)	**3.9 (2.0, 5.7)**
	RIS	−1.2 (−3.3, 0.9)	−1.9 (−4.9, 1.0)	−0.9 (−2.6, 0.8)	−1.1 (−2.7, 0.6)
	DMAb‐RIS	3.5 (0.4, 6.6)*	**7.9 (3.5, 12.2)†**	2.6 (−0.1, 5.2)	**4.9 (2.4, 7.5)†**
Cortical volume	DMAb	1.8 (−0.1, 3.8)	**3.1 (0.5, 5.6)**	1.2 (−1.1, 3.6)^a^	**5.0 (1.8, 8.1)**
	RIS	−0.6 (−2.5, 1.4)^a^	−1.8 (−4.3, 0.8)^b^	−1.0 (−3.0, 1.1)^e^	**−2.9 (−5.7, −0.1)**
	DMAb‐RIS	2.4 (−0.4, 5.2)	4.8 (1.1, 8.6)*	2.2 (−1.0, 5.4)	**7.9 (3.5, 12.3)†**
Ct.BMD	DMAb	−0.3 (−1.5, 1.0)	**1.8 (0.5, 3.1)**	0.3 (−0.8, 1.3)	0.8 (−0.2, 1.7)
	RIS	**−1.7 (−2.9, −0.6)**	**−2.3 (−3.5, −1.0)**	**−1.6 (−2.6, −0.6)**	**−1.5 (−2.4, −0.7)**
	DMAb‐RIS	1.5 (−0.2, 3.2)	**4.1 (2.3, 5.9)†**	1.9 (0.4, 3.3)*	**2.3 (1.0, 3.6)†**
Ct.Th	DMAb	2.7 (−0.8, 6.2)	**6.4 (1.9, 10.9)**	**3.3 (0.9, 5.7)**	3.3 (−0.6, 7.3)
	RIS	−2.5 (−5.7, 0.8)	−3.6 (−7.9, 0.7)	−1.2 (−3.2, 0.9)	−1.8 (−5.3, 1.6)
	DMAb‐RIS	5.2 (0.3, 10.0)*	10.0 (3.5, 16.5)*	4.5 (1.2, 7.8)*	5.2 (−0.7, 11.0)
Ct.Po	DMAb	5.9 (−2.8, 14.7)^c^	0.6 (−15.7, 16.8)^d^	−0.5 (−6.8, 5.9)^a^	6.1 (−1.1, 13.3)
	RIS	0.3 (−8.2, 8.7)^a^	14.1 (−1.4, 29.7)^b^	2.3 (−3.2, 7.9)^e^	−3.5 (−10.0, 3.0)
	DMAb‐RIS	5.7 (−6.5, 17.8)	−13.6 (−36.3, 9.2)	−2.8 (−11.2, 5.6)	9.6 (−0.1, 19.3)
Tb.BMD	DMAb	1.4 (−2.4, 5.2)	**4.7 (0.0, 9.4)**	0.4 (−1.9, 2.6)	**3.1 (1.1, 5.1)**
	RIS	−2.0 (−5.5, 1.6)	−2.1 (−6.6, 2.4)	−0.7 (−2.7, 1.3)	−0.2 (−2.0, 1.6)
	DMAb‐RIS	3.4 (−1.8, 8.6)	6.8 (0.3, 13.3)*	1.1 (−2.0, 4.1)	3.3 (0.6, 6.0)*
Tb.BV/TV	DMAb	1.9 (−1.2, 5.1)	**5.3 (1.5, 9.2)**	0.9 (−1.4, 3.1)	**3.7 (1.5, 5.8)**
	RIS	0.3 (−2.6, 3.3)	0.2 (−3.5, 3.9)	0.8 (−1.2, 2.8)	1.3 (−0.7, 3.2)
	DMAb‐RIS	1.6 (−2.7, 5.9)	5.1 (−0.3, 10.5)	0.1 (−2.9, 3.1)	2.4 (−0.6, 5.3)
Tb.N	DMAb	3.7 (−1.1, 8.4)	**5.9 (1.0, 10.7)**	−0.6 (−6.3, 5.0)	2.7 (−4.8, 10.2)
	RIS	−1.4 (−5.9, 3.1)	−1.0 (−5.7, 3.7)	−1.9 (−6.9, 3.1)	1.8 (−4.9, 8.6)
	DMAb‐RIS	5.1 (−1.5, 11.6)	6.9 (0.1, 13.7)*	1.2 (−6.4, 8.8)	0.8 (−9.3, 11.0)
Tb.Th	DMAb	−1.0 (−5.3, 3.4)	1.2 (−3.9, 6.4)	4.0 (−1.8, 9.9)	3.8 (−1.8, 9.3)
	RIS	1.5 (−2.6, 5.5)	0.5 (−4.5, 5.4)	2.8 (−2.4, 8.0)	−0.7 (−5.7, 4.3)
	DMAb‐RIS	−2.4 (−8.4, 3.5)	0.8 (−6.4, 8.0)	1.2 (−6.7, 9.2)	4.4 (−3.2, 12.0)
Tb.Sp	DMAb	−3.2 (−8.0, 1.5)	**−5.0 (−9.6, −0.3)**	1.8 (−4.2, 7.9)	−1.3 (−8.5, 5.8)
	RIS	1.8 (−2.7, 6.3)	1.3 (−3.2, 5.7)	2.7 (−2.7, 8.1)	−0.7 (−7.1, 5.7)
	DMAb‐RIS	−5.0 (−11.6, 1.5)	−6.2 (−12.7, 0.2)	−0.9 (−9.0, 7.3)	−0.6 (−10.3, 9.0)

DMAb = denosumab; RIS = risedronate; Tt.BMD = total bone mineral density; Ct.BMD = cortical bone mineral density; Ct.Th = cortical thickness; Ct. Po = cortical porosity; Tb.BMD = trabecular bone mineral density; Tb.BV/TV = trabecular bone volume fraction; Tb.N = trabecular number; Tb.Th = trabecular thickness; Tb.Sp = trabecular separation.

Data are reported as least‐square means (95% confidence interval).

*N* = number of randomized patients enrolled in the HR‐pQCT substudy; *n*_D_ = number of patients with observed data receiving denosumab; *n*_R_ = number of patients with observed data receiving risedronate.

^a^ *n*_R_ = 14 and/or *n*_D_ = 14.

^b^ *n*_R_ = 12.

^c^ *n*_D_ = 13.

^d^ *n*_D_ = 11.

^e^ *n*_R_ = 18.

Percentage changes with and differences between the treatment groups are based on repeated‐measures mixed‐effects models adjusted for treatment, baseline value, and duration of prior GC‐use (<12 months versus ≥12 months). Significant changes within and differences between the treatment groups are reported in bold (*p* < 0.05). *p < 0.05 and †*p* ≤ 0.001 for differences between the treatment groups.

### Evaluation of BMD and bone microarchitecture

In GC‐I, total BMD (Tt.BMD) significantly increased with denosumab at the radius at 12 months (1.2%, 95% CI 0.0, 2.3) and 24 months (2.3%, 95% CI 0.8, 3.7), whereas it remained unchanged with risedronate (Table 4 and Fig. 1). Corresponding differences between denosumab and risedronate at the radius were significant (12 months: 2.1%, *p* = 0.015; 24 months: 3.7%, *p* < 0.001). At the tibia, it remained unchanged with denosumab, whereas it significantly decreased with risedronate at 24 months (−1.8%, 95% CI −3.1, −0.5), leading to a significant between‐treatment difference at this time point (2.9%, *p* = 0.002).

Significant changes and differences were also found in the cortical bone compartment (Table 4 and Fig. 1). Cortical BMD (Ct.BMD) remained unchanged at 12 months at the radius and tibia with denosumab and risedronate, whereas at 24 months, it significantly changed at the tibia (denosumab: 1.0%, 95% CI 0.2, 1.8; risedronate: −1.1%, 95% CI −1.9, −0.2) with corresponding significant between‐treatment difference (2.1%, *p* < 0.001). Although it did not change significantly at the radius at 24 months, corresponding between‐treatment difference did (1.4%, *p* = 0.027). Cortical thickness (Ct.Th) showed more pronounced changes. At the radius, it significantly increased with denosumab at 12 and 24 months (2.1%, 95% CI 0.4, 3.8, and 2.8%, 95% CI 0.3, 5.2, respectively), whereas it significantly decreased with risedronate (12 months: −2.4%, 95% CI −4.2, −0.7; 24 months: −4.0%, 95% CI −6.6, −1.4), resulting in significant between‐treatment differences (12 months: 4.6%, *p* < 0.001; 24 months: 6.7%, *p* < 0.001). At the tibia, Ct.Th remained unchanged with denosumab, whereas it significantly decreased with risedronate at 24 months (−3.7%, 95% CI −5.9, −1.6), leading to a significant difference between denosumab and risedronate at 24 months (5.5%, *p* < 0.001) but also at 12 months (2.5%, *p* = 0.024). Cortical porosity (Ct.Po) remained unchanged with denosumab and risedronate at both time points and scan locations except for a significant increase at 24 months with denosumab at the radius and with risedronate at the tibia.

Less prominent changes and differences were found in the trabecular bone compartment (Table 4). Trabecular BMD (Tb.BMD) remained unchanged at 12 months with denosumab and risedronate and significantly changed at 24 months with denosumab (radius: 3.4%, 95% CI 1.5, 5.3) but not with risedronate, resulting in a significant difference between denosumab and risedronate at this time point (radius: 3.3%, *p* = 0.020). Similarly, trabecular bone volume fraction (Tb.BV/TV), number (Tb.N), thickness (Tb.Th), and separation (Tb.Sp) remained unchanged with denosumab and risedronate at 12 and 24 months except with denosumab at 24 months at the radius in Tb.BV/TV, Tb.N, and Tb.Sp and at the tibia in Tb.BV/TV, but these changes did not lead to significant differences between denosumab and risedronate.

In GC‐C, Tt.BMD significantly increased with denosumab at 12 months (radius: 2.3%, 95% CI 0.0, 4.5) and 24 months (radius: 5.9%, 95% CI 2.9, 9.0; tibia: 3.9%, 95% CI 2.0, 5.7), whereas it remained unchanged at both time points with risedronate (Table 5 and Fig. 1). Corresponding differences between denosumab and risedronate were significant at 12 months (radius: 3.5%, *p* = 0.030) and 24 months (radius: 7.9%, *p* = 0.001; tibia: 4.9%, *p* < 0.001).

Similar to GC‐I, compartmental changes and differences in GC‐C were mainly found in the cortical bone (Table 5 and Fig. 1). At 12 months, Ct.BMD remained unchanged with denosumab and significantly decreased with risedronate (radius: −1.7%, 95% CI −2.9, −0.6; tibia: −1.6%, 95% CI −2.6, −0.6), resulting in a significant between‐treatment difference at the tibia (1.9%, *p* = 0.014) but not at the radius. At 24 months, Ct.BMD significantly increased with denosumab (radius: 1.8%, 95% CI 0.5, 3.1) and significantly decreased with risedronate (radius: −2.3%, 95% CI −3.5, −1.0; tibia: −1.5%, 95% CI −2.4, −0.7) with corresponding significant between‐treatment differences (radius: 4.1%, *p* < 0.001; tibia: 2.3%, *p* = 0.001). Ct.Th in contrast did not change significantly with risedronate at 12 months at the radius and tibia but significantly increased with denosumab at the tibia (3.3%, 95% CI 0.9, 5.7), resulting in a significant between‐treatment difference (4.5%, *p* = 0.010). Although the changes at the radius did not reach significance at 12 months, the between‐treatment difference did (5.2%, *p* = 0.038). At 24 months, Ct.Th remained unchanged with risedronate, whereas it significantly increased with denosumab at the radius (6.4%, 95% CI 1.9, 10.9) with corresponding significant difference between denosumab and risedronate (10.0%, *p* = 0.004). No significant changes and differences were found in Ct.Po.

Less pronounced changes and differences were found in the trabecular bone compartment (Table 5). Tb.BMD remained unchanged at 12 months with denosumab and risedronate and significantly changed at 24 months with denosumab (radius: 4.7%, 95% CI 0.0, 9.4; tibia: 3.1%, 95% CI 1.1, 5.1) and not with risedronate, resulting in significant differences between denosumab and risedronate at 24 months (radius: 6.8%, *p* = 0.042; tibia: 3.3%, *p* = 0.018). Similar as in GC‐I, no significant changes with and differences between denosumab and risedronate were found in the trabecular microarchitecture parameters except for a significant change at 24 months with denosumab at the radius in Tb.BV/TV, Tb.N, and Tb.Sp and at the tibia in Tb.BV/TV with a corresponding just significant between‐treatment difference in Tb.N at the radius.

## Discussion

The aim of this HR‐pQCT study was to assess changes in cortical and trabecular microarchitecture and estimated bone strength during 24 months with denosumab compared with risedronate in GC‐I and GC‐C. We found that FL was maintained with denosumab at the radius and tibia in GC‐I at 24 months, whereas it decreased with risedronate at the radius, leading to a significant difference in FL at the radius and tibia in favor of denosumab. In GC‐C at 24 months, FL significantly increased with denosumab at the radius, whereas it was maintained with risedronate, resulting in a significant difference in FL in favor of denosumab at the radius.

In GC‐I, maintenance of FL at the radius at 24 months with denosumab coincided with an increase in Ct.Th, which is compatible with the rapid reduction of bone resorption, filling of existing resorption sites, and appearance of fewer newly excavated sites at the endosteum, similar to a previous report of denosumab in PMOP.^(^ ^9^ ^)^ Simultaneously, Ct.BMD remained unchanged, which indicates that the expected loss in Ct.BMD^(^ ^7^, ^8^ ^)^ due to increased intracortical bone remodeling after initiating high‐dose GC^(^ ^24^ ^)^ did not occur with denosumab. Ct.Po increased at 24 months, which, together with the unchanged Ct.BMD, could be the result of the inclusion of highly mineralized trabeculae in the endosteal border of the cortex. With risedronate, in contrast, FL at the radius decreased at 24 months, which coincided with a decrease in Ct.Th and consequent significant difference from denosumab (in favor of denosumab). This effect on Ct.Th indicates that risedronate, as opposed to denosumab, could not halt endosteal bone loss in GC‐I, which is the result of increased osteoclast activity by GCs,^(^ ^25^ ^)^ also at the endosteal site of the cortex.^(^ ^26^, ^27^ ^)^ Different effects were found at the tibia, eg, with denosumab, FL was also maintained at the tibia at 24 months but with an increase in Ct.BMD and not in Ct.Th, and with risedronate, Ct.BMD was not maintained at the tibia as was found at the radius but decreased. The latter may be explained by the coinciding increase in Ct.Po at the tibia, which indicates that intracortical bone remodeling remained unchanged with risedronate at the radius but not at the tibia. The differences between the radius and tibia suggest a role of the loading of bones, which can be affected by the suppressive effect of GCs on osteocytes and osteoblasts.^(^ ^28^ ^)^

Interestingly, different treatment effects were found in GC‐C compared with GC‐I. In GC‐C, FL increased at the radius at 24 months with denosumab, which coincided with an increase in Ct.Th and Ct.BMD. At the tibia, these parameters were maintained with denosumab. With risedronate, FL was also maintained but coincided with a decrease in Ct.BMD. This implies that risedronate could not prevent the loss of Ct.BMD with long‐term GC,^(^ ^7^, ^8^ ^)^ as opposed to denosumab. Ct.Th was maintained with risedronate at both scan sites and both time points, which contrasts with the decrease observed in GC‐I.

In both subpopulations, few changes and differences were found with and between denosumab and risedronate in the trabecular bone compartment. At 24 months, Tb.BMD and Tb.N increased with denosumab at the radius in GC‐I and at radius and tibia in GC‐C, whereas these parameters were maintained with risedronate, leading to a significantly higher percentage change between denosumab and risedronate. These changes indicate that trabecular bone was preserved with risedronate and increased with denosumab, which is in line with changes in aBMD in the spine and trochanter with denosumab and risedronate.^(^ ^5^ ^)^ Additionally, the increase in Tb.BMD in GC‐I and GC‐C with denosumab, together with the changes in Ct.BMD, contributed to a significant increase in Tt.BMD in both subpopulations, which was significantly higher than the changes with risedronate.

The larger effect of denosumab compared with risedronate on bone strength among GC‐C users in this study, who had low BMD *T*‐score at baseline, agrees with previous findings in postmenopausal women with low BMD. Using QCT, Genant and colleagues showed significant and progressive increases in polar moment of inertia (PMI), a measure for bone strength, at the ultradistal, distal, and proximal radius starting 6 months after treatment with denosumab.^(^ ^12^ ^)^ Seeman and colleagues found similar increases in PMI at the distal radius with denosumab and reported this effect to be superior over that with alendronate.^(^ ^9^ ^)^ They found, however, an increase in PMI and a maintained Ct.BMD with alendronate, whereas we found a maintained FL and a decrease in Ct.BMD with risedronate. This difference between risedronate and alendronate agrees with previously reported larger gains in lumbar spine, femoral neck, and total hip aBMD at 24 months with alendronate than with risedronate in postmenopausal women with low BMD without GC‐use.^(^ ^29^ ^)^ Interestingly, the results for Tt.BMD were in general similar to those for FL. Further studies are needed to analyze which bone parameters influence changes in FL in GC users and to what degree.

Aside from Tt.BMD (GC‐C) and Tb.BMD (GC‐I and GC‐C), the changes in FL and stiffness and multiple other HR‐pQCT parameters were less than pooled least significant changes (LSCs) at the individual level^(^ ^30^ ^)^; nevertheless, they may be clinically relevant at the group level in clinical trials. Although the LSC of total hip BMD in postmenopausal women has been found to be approximately 4.5%,^(^ ^31^, ^32^ ^)^ a recent meta‐analysis of placebo‐controlled clinical trials on anti‐osteoporosis drugs showed that an increase in mean total hip BMD of 2% was associated with a decrease in vertebral and hip fracture risk of 28% and 16%, respectively.^(^ ^33^ ^)^ Furthermore, a difference in mean change between treatment and placebo in total hip BMD of more than 1.4%, 3.2%, and 2.1% was associated with risk reductions of vertebral, hip, and non‐vertebral fractures, respectively.^(^ ^34^ ^)^ The authors concluded that these levels of mean BMD changes over time and differences between groups support BMD as a surrogate outcome for fracture outcomes at the group level in randomized trials of new osteoporosis therapies.^(^ ^33^, ^34^ ^)^ By analogy, in Tt.BMD, we found significant changes with denosumab of 1.2% to 5.9% and differences between denosumab and risedronate of 2.1% to 7.9%, which are similar to or exceed these changes in total hip BMD. Besides that, mean HR‐pQCT parameters at the group level have been found to improve fracture prediction beyond femoral neck BMD alone, with FL having the strongest association.^(^ ^35^ ^)^ Also in FL, we found significant changes with denosumab and differences between denosumab and risedronate that were similar to or larger than these clinically relevant changes in total hip BMD. In analogy with the studies of Bouxsein and colleagues^(^ ^33^ ^)^ and Black and colleagues^(^ ^34^ ^)^ on the use of DXA as a surrogate marker for the effect on fractures, further study is needed to investigate the association between the level of changes and between‐treatment differences in HR‐pQCT parameters on the one hand and the effect on fracture risk on the other hand to better elucidate the clinical relevance of changes in the HR‐pQCT parameters with anti‐osteoporosis drugs.^(^ ^36^ ^)^ Also, the correlation between changes and differences in FL with changes and differences in other HR‐pQCT parameters requires further study.

The findings of the current study can contribute to treatment decisions for fracture prevention in GC‐I and GC‐C. With only limited fracture data from clinical trials on GIOP, fracture reduction with GIOP treatments is mostly based on an extrapolation of fracture risk reductions ascertained in clinical trials on treatments of osteoporosis in general.^(^ ^37^ ^)^ In clinical trials in postmenopausal women, changes in lumbar spine, femoral neck, and total hip aBMD are considered a useful surrogate endpoint for fracture.^(^ ^33^ ^)^ However, the relation between aBMD and fracture risk is different in GIOP than PMOP, with a higher fracture risk in GC users than nonusers with the same aBMD.^(^ ^14^, ^15^, ^16^, ^17^, ^18^, ^19^, ^37^ ^)^ HR‐pQCT parameters and FL improve fracture prediction beyond femoral neck aBMD.^(^ ^35^ ^)^ In GC‐I, with only moderately low *T*‐scores, the aim of fracture prevention is then to preserve FL, whereas in GC‐C, with considerably lower *T*‐scores reflecting already mechanically compromised bone, the aim is to increase FL. As such, HR‐pQCT assessment may give additional insights into bone strength in clinical trials that are not powered to evaluate fracture prevention, especially when limited study populations can be included, such as in GC‐I and GC‐C.

Both aims were attained by denosumab but not by risedronate in this study, but awareness is needed regarding desired therapeutic option. For example, the effects of drug discontinuation require attention. In contrast to risedronate and other bisphosphonates, denosumab is not incorporated into the bone matrix. Correspondingly, recent data indicate an increased bone turnover after discontinuation of denosumab resulting in rapid BMD loss and an increased risk of multiple vertebral fractures.^(^ ^38^ ^)^ It is therefore important that treatment with denosumab should not be stopped without considering alternative antiresorptive treatment.^(^ ^39^ ^)^ Another possible difference between anti‐osteoporosis drugs that requires attention is the adherence rate due to differences in drug administration. Nevertheless, in this study, the adherence rate was high for both oral and injectable administration: Based on the determination of “important protocol deviations” after medical review by the original study site and team, only 8.2% and 0.9% missed >20% of an oral product (placebo or risedronate) during the first 12 and 24 months, respectively, and none of the patients missed an injectable (placebo or denosumab) during the entire study duration.

Despite the strengths and its novelty, this study has several limitations. First, data were obtained using the first‐generation HR‐pQCT scanner, which limits direct measurement of trabecular microarchitecture to Tb.N and the quantification of Ct.Po to pores larger than the scan resolution.^(^ ^20^ ^)^ Use of the second‐generation HR‐pQCT scanner with higher resolution would have allowed more detailed assessment of trabecular microarchitecture and Ct.Po and may have an improved reproducibility but was not available in the participating centers. Second, only standard HR‐pQCT parameters were quantified. However, BMD is influenced by void volumes, whereas volumetric tissue mineral density purely examines mineralized bone. The latter parameter may therefore provide better understanding of the combined effects of denosumab on Ct.BMD, Ct.Th, and Ct.Po (eg, the increase in Ct.BMD in GC‐C at 24 months without a coinciding decrease in Ct.Po) but was not calculated. Furthermore, the standard parameters do not provide detailed insights into endosteal changes. For example, the unchanged total bone volume and increased Ct.Th with denosumab at the radius at 24 months in GC‐C suggest that the corresponding increase in FL could be the result of endocortical changes. However, quantification of such changes requires more advanced analysis methods, such as the recent method to separately segment compact‐appearing cortical bone and cortical transitional zones to evaluate cortical parameters in greater detail in each segment.^(^ ^40^ ^)^ Third, we indicated the significance of changes from baseline and for differences between treatment groups without correction for multiplicity, as in other studies reporting the multiple parameters generated with HR‐pQCT during treatment versus placebo or between drugs (eg, Seeman and colleagues^(9)^ and Tsai and colleagues^(10)^). When using a false discovery rate (FDR)‐based adjustment to correct for multiple testing to reject false positive results, we found that the differences between denosumab and risedronate at 24 months remained significant at the radius for FL, stiffness, Tt.BMD, cortical volume, and Ct.Th in GC‐I and for Tt.BMD, Ct.BMD, and Ct.Th in GC‐C, and at the tibia for Tt.BMD, Ct.BMD, and Ct.Th in GC‐I and for Tt.BMD, cortical volume, and Ct.BMD in GC‐C. Trabecular parameters were not significantly different any more between treatment groups after FDR adjustment.

In conclusion, in this HR‐pQCT study, we found that denosumab was superior to risedronate in terms of preventing FL and Tt.BMD loss in GC‐I at the radius and tibia and in increasing FL and Tt.BMD in GC‐C at the radius. We also identified underlying differences in changes in the cortical and trabecular bone compartments between denosumab and risedronate in GC‐I and GC‐C. These results suggest that denosumab could be a useful therapeutic option in patients initiating GC therapy or on long‐term GC therapy and may contribute to treatment decisions in this patient population.

## Disclosures

PG reports grants from Amgen, grants and other from AbbVie, grants from Celgene, grants from Lilly, grants from Merck, grants from Pfizer, grants from Roche, grants from UCB, grants from Fresenius, grants from Mylan, and grants from Sandoz outside the submitted work. BR reports personal fees from Scanco Medical AG outside the submitted work. EL reports personal fees from Amgen; personal fees from Lilly; grants, personal fees, and non‐financial support from Celgene; personal fees from Expanscience; personal fees from AbbVie; personal fees from Sublimed; grants, personal fees, and non‐financial support from UCB; and grants, personal fees, and non‐financial support from MSD outside the submitted work. BO reports personal fees from Raffo, personal fees from Baliarda, personal fees from Shire, personal fees from Takeda, personal fees from Gador, personal fees from Amgen, and personal fees from Ultragenyx outside the submitted work. RC reports grants from Amgen during the conduct of the study; grants and personal fees from UCB; personal fees from Lilly; personal fees from BMS; personal fees from AbbVie; personal fees from Pfizer; grants and personal fees from Chugai; personal fees from Sanofi; personal fees from Novartis; and grants and other from Fresenius Kiabi outside the submitted work. AC is employed by Amgen and has equity in Amgen. SH is an employee of and has equity in Amgen. KGS reports grants from Amgen, Horizon Pharma, Swedish Orphan Biovitrum AB, Shanton Pharma Co. LTD, Dyve Bioscience, and LG Chem outside the submitted work. JPB reports grants from Amgen, grants from UCB, and grants from Lilly the outside the submitted work. All authors had full access to the data, and there were no restrictions regarding content to include in the manuscript.

## Author Contributions

**Piet Geusens:** Investigation; writing – original draft; writing – review and editing. **Melissa Bevers:** Writing – original draft; writing – review and editing. **Bert van Rietbergen:** Writing – review and editing. **Osvaldo Daniel Messina:** Investigation; writing – review and editing. **Eric Lespessailles:** Investigation; writing – review and editing. **Beatriz Oliveri:** Investigation; writing – review and editing. **Roland Chapurlat:** Investigation; writing – review and editing. **Klaus Engelke:** Formal analysis; writing – review and editing. **Arkadi Chines:** Formal analysis; writing – review and editing. **Shuang Huang:** Formal analysis; writing – review and editing. **Kenneth Saag:** Investigation; writing – review and editing. **Joop van den Bergh:** Writing – review and editing.

## Data Availability

This study was funded by Amgen Inc. Qualified researchers may request data from Amgen clinical studies. Complete details are available at the following: https://wwwext.amgen.com/science/clinical-trials/clinical-data-transparency-practices/clinical-trial-data-sharing-request/.
